# Diagnostic delay of acute mitral regurgitation during the coronavirus disease 2019 pandemic: a case report

**DOI:** 10.1186/s12245-021-00365-6

**Published:** 2021-07-19

**Authors:** Azumi Kawabata, Hiraku Funakoshi, Joji Ito, Takushi Santanda, Yasuhiro Norisue, Hiroyuki Watanabe

**Affiliations:** 1 Department of Emergency and Critical Care Medicine, Tokyo Bay Urayasu Ichikawa Medical Center, 3-4-32, Todaijima, Urayasu, Chiba, 279-0001 Japan; 2 Department of Cardiovascular Surgery, Tokyo Bay Urayasu Ichikawa Medical Center, 3-4-32, Todaijima, Urayasu, Chiba, 279-0001 Japan; 3 Department of Cardiology, Tokyo Bay Urayasu Ichikawa Medical Center, 3-4-32, Todaijima, Urayasu, Chiba, 279-0001 Japan

**Keywords:** Bias, COVID-19, Diagnostic error, Dyspnea, Mitral valve prolapse

## Abstract

**Background:**

Diagnostic errors or delays can cause serious consequences for patient safety, especially in the emergency department. Anchoring bias is one of the major factors leading to diagnostic error. During the coronavirus disease 2019 (COVID-19) pandemic, the high probability of COVID-19 in febrile patients could be a major cause of anchoring bias leading to diagnostic error. In addition, certain evaluations such as auscultation are difficult to perform on a casual basis due to the increased risk of contact infection, which lead to inadequate assessment of the patients with valvular disease.

Acute mitral regurgitation (MR) could be a fatal disease in the emergency department, especially if there is a diagnostic error or delay in diagnosis. It is often reported that diagnosis can be difficult even though there is no treatment other than emergent surgery.

The diagnosis of acute MR has become more difficult because coronavirus disease 2019 (COVID-19) pandemic could affect our daily practice especially in febrile patients.

We report a case of a diagnostic delay of a febrile patient because of anchoring bias during the COVID-19 pandemic.

**Case presentation:**

A 45-year-old man presented to the emergency department complaining of acute dyspnea and fever. Based on vital signs and computed tomography of the chest, acute pneumonia due to COVID-19 was suspected. Auscultation was avoided because of facility rule based on concern of contact infection. After admission to the intensive care unit, Doppler echocardiography revealed acute mitral regurgitation, and transesophageal echocardiography revealed mitral valve tendon rupture. After confirming the negative result for the polymerase chain reaction of severe acute respiratory syndrome coronavirus 2, mitral valvuloplasty was performed on the third day after admission. The patient was discharged 14 days after admission without complications.

**Conclusions:**

In COVID-19 pandemic, anchoring bias suspecting COVID-19 among febrile patients becomes a strong heuristic factor. A thorough history and physical examination is still important in febrile patients presenting with dyspnea to ensure the correct diagnosis of acute mitral regurgitation.

**Supplementary Information:**

The online version contains supplementary material available at 10.1186/s12245-021-00365-6.

## Background

Diagnostic errors and delays are of serious concern for patient safety, and more common in the emergency department (ED) than in other departments [1, 2]. Although the ED is a challenging environment in which to make a timely and accurate diagnosis, diagnostic delays in the ED could lead to unstable conditions, unnecessary procedures, or even be fatal [3, 4].

The coronavirus disease 2019 (COVID-19) pandemic affects the daily practice of physicians in many aspects; emergency physicians must pay more attention to infection control and wear personal protective equipment (PPE) during work shifts, and some evaluations including auscultation are difficult to perform casually because of concerns about contact infection. In addition, the high probability of COVID-19 being present in febrile patients during the pandemic could lead an anchoring bias that cause diagnostic error or delay [5].

Despite these factors, reports of diagnostic errors related to the COVID-19 pandemic have been scarce. Hereby, we report the case of a febrile patient with acute mitral regurgitation, whose diagnosis was delayed due to several factors related to the COVID-19 pandemic.

## Case presentation

A 45-year-old man with an unremarkable past medical history was transported to our hospital complaining of acute onset of dyspnea and fever during the COVID-19 pandemic. The patient had been healthy until going to bed. Several hours later, the patient awoke with a feeling of breathlessness; an ambulance was called when there was no sign of improvement.

The patient was an ex-smoker, and had been taking no medications. On examination, the patient was alert and fully oriented; his temperature was 38.1 °C, blood pressure was 146/80 mmHg, pulse rate was 117 beats per minute, respiratory rate was 30 breaths per minute, and oxygen saturation was 90% with 15 L oxygen via a mask with a non-rebreathing reservoir. The patient could not lie on his back, and was breathing in a sitting position and diaphorating. There was no jugular vein dilatation or peripheral edema.

**Fig. 1:**
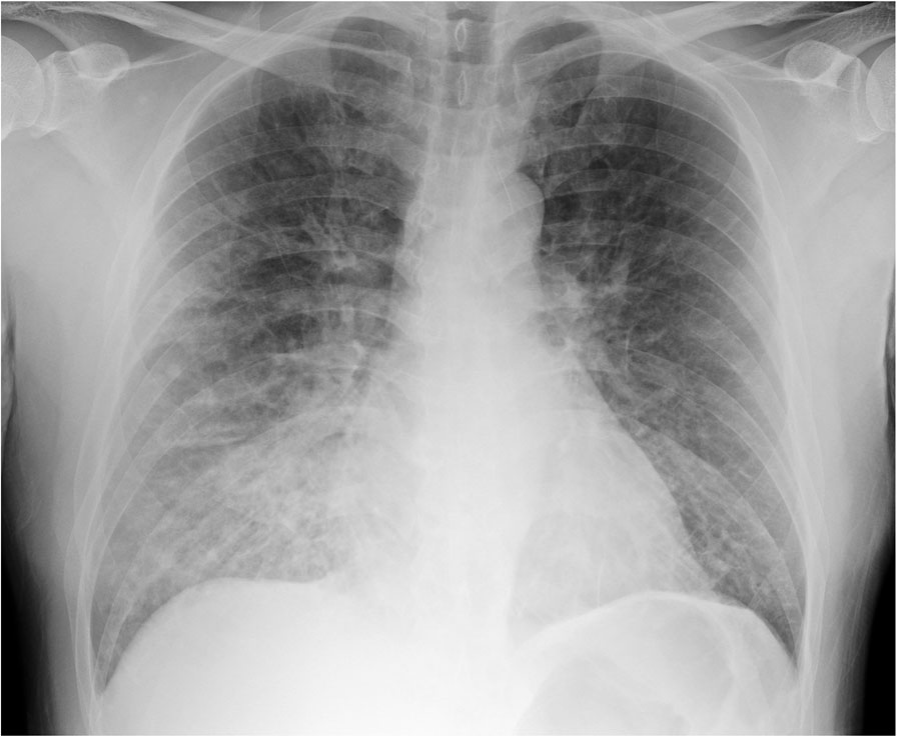
Chest X-ray revealing indistinct pulmonary vessels, diffuse opacities in the lower lung zones, which were more prominent in the right lung

**Fig. 2:**
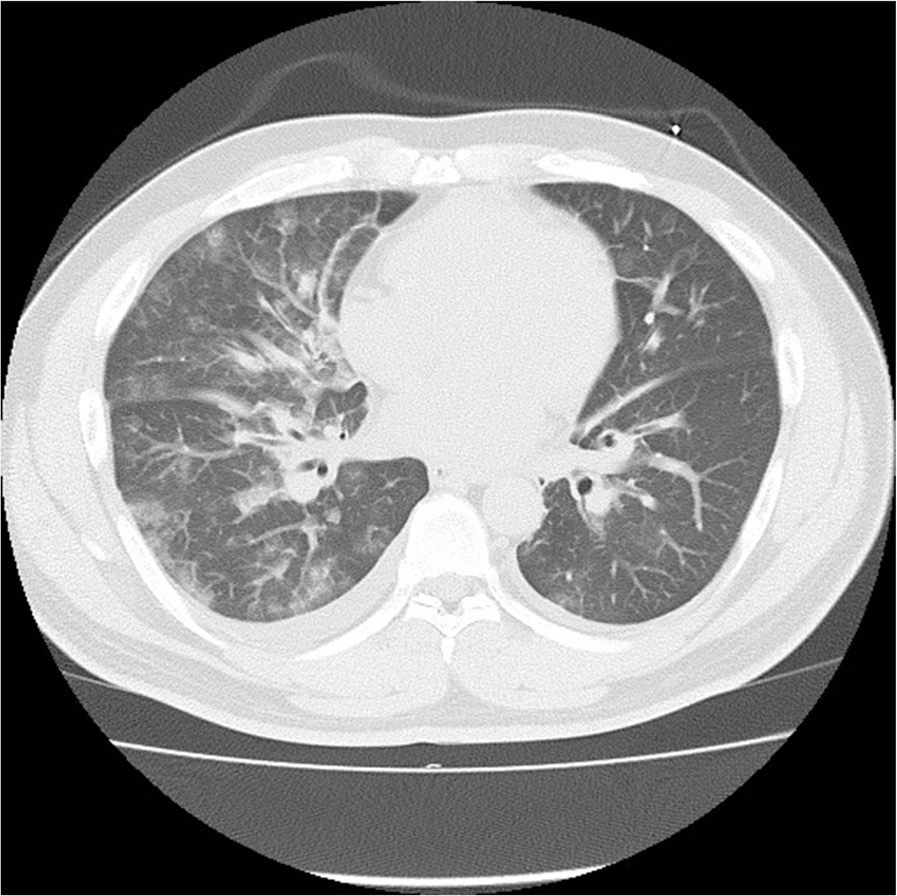
Chest computed tomography showed ground glass opacity with diffuse lobular and septal wall thickening

Due to concerns about COVID-19 transmission, the patient was examined with full PPE under negative pressure from the beginning of his treatment, where auscultation was avoided because of facility rule based on concern of contact infection. The laboratory data showed no significant changes except for mild elevation of lactate and high-sensitivity troponin I (Table 1). Portable chest X-ray showed no typical pulmonary edema, but indicated decreased permeability predominantly in the right lung (Fig. 1). Point of care ultrasound (POCUS) revealed bilateral multiple B-lines and hyperdynamic contractions, which showed a visual ejection fraction (EF) of 50% and no regional wall motion abnormality. On the echocardiogram (ECG), no ST changes, negative T waves, or arrhythmias were noted. Computed tomography of the chest showed ground glass opacity with diffuse lobular septal wall thickening (Fig. 2).

**Table 1:** Laboratory data on arrival

Variable	On arrival
Hematocrit (%)	49
Hemoglobin (g/dL)	17.5
White-cell count (per mm^3^)	18200
Erythrocyte count (per mm^3^)	5,400,000
Platelet count (per mm^3^)	29.3
Sodium (mEq/L)	144
Potassium (mEq/L)	4
Chloride (mEq/L)	105
Urea nitrogen (mg/dL)	25.5
Creatinine (mg/dL)	1.17
Albumin (g/dL)	4.3
Creatine kinase (U/L)	167
CK-MB (U/L)	5.5
Troponin I (ng/mL)	0.079
C-reactive protein (mg/dL)	0.38
Brain natriuretic peptide (pg/mL)	256.9
**Blood gases (vein)**	
pH	7.314
Partial pressure of carbon dioxide (mmHg)	52.4
Partial pressure of oxygen (mmHg)	33.4
Base excess	−1
Lactate (mg/dL)	22

Based on the history of acute onset respiratory failure without respiratory symptoms, the patient was suspected to have acute hypertensive heart failure. However, because of the pandemic, COVID-19 could not be ruled out as the cause of respiratory failure.

**Fig. 3:**
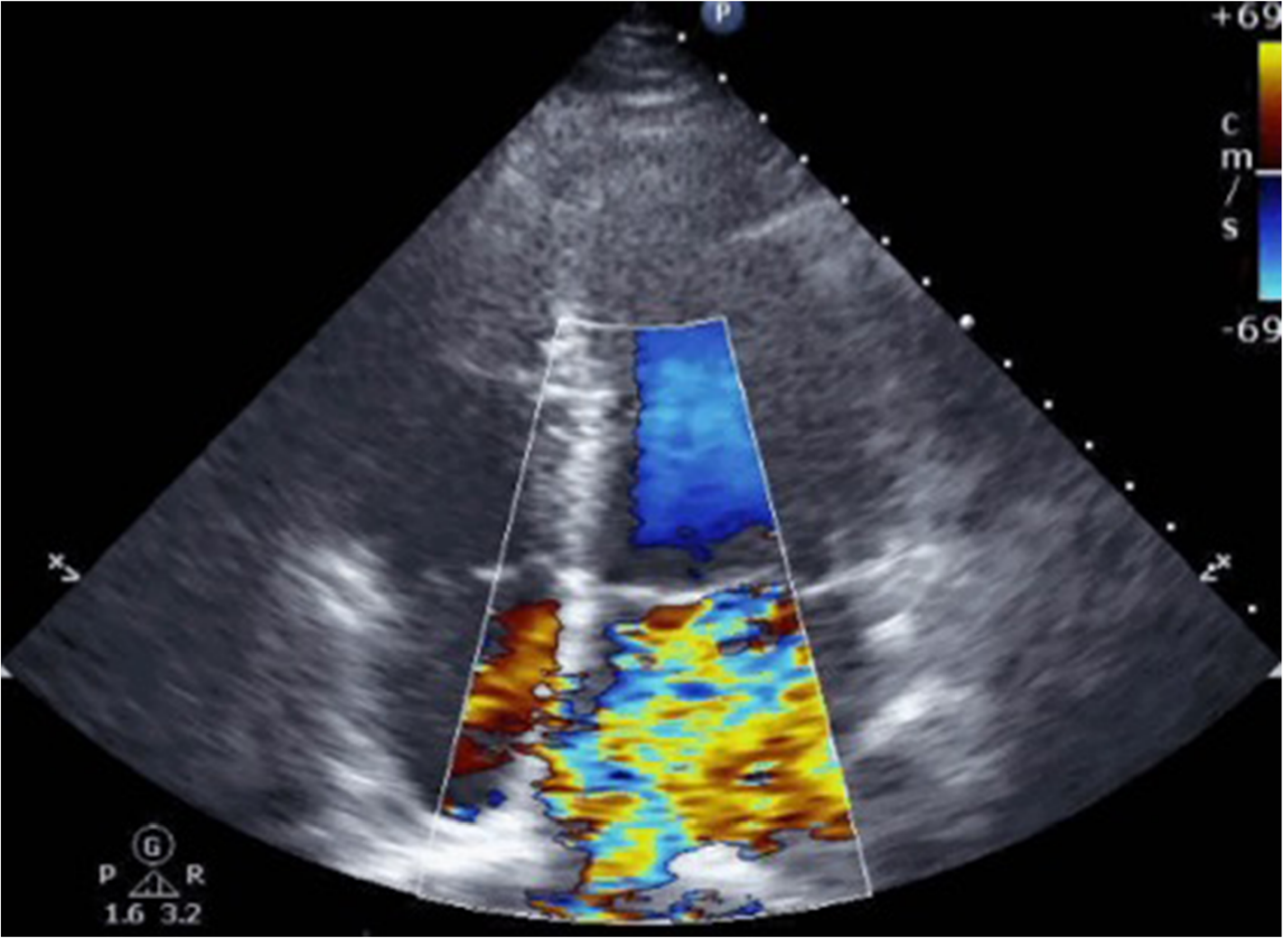
Transthoracic echocardiography with Doppler revealed a severe mitral valve regurgitation from the apical four chamber view

**Fig. 4:**
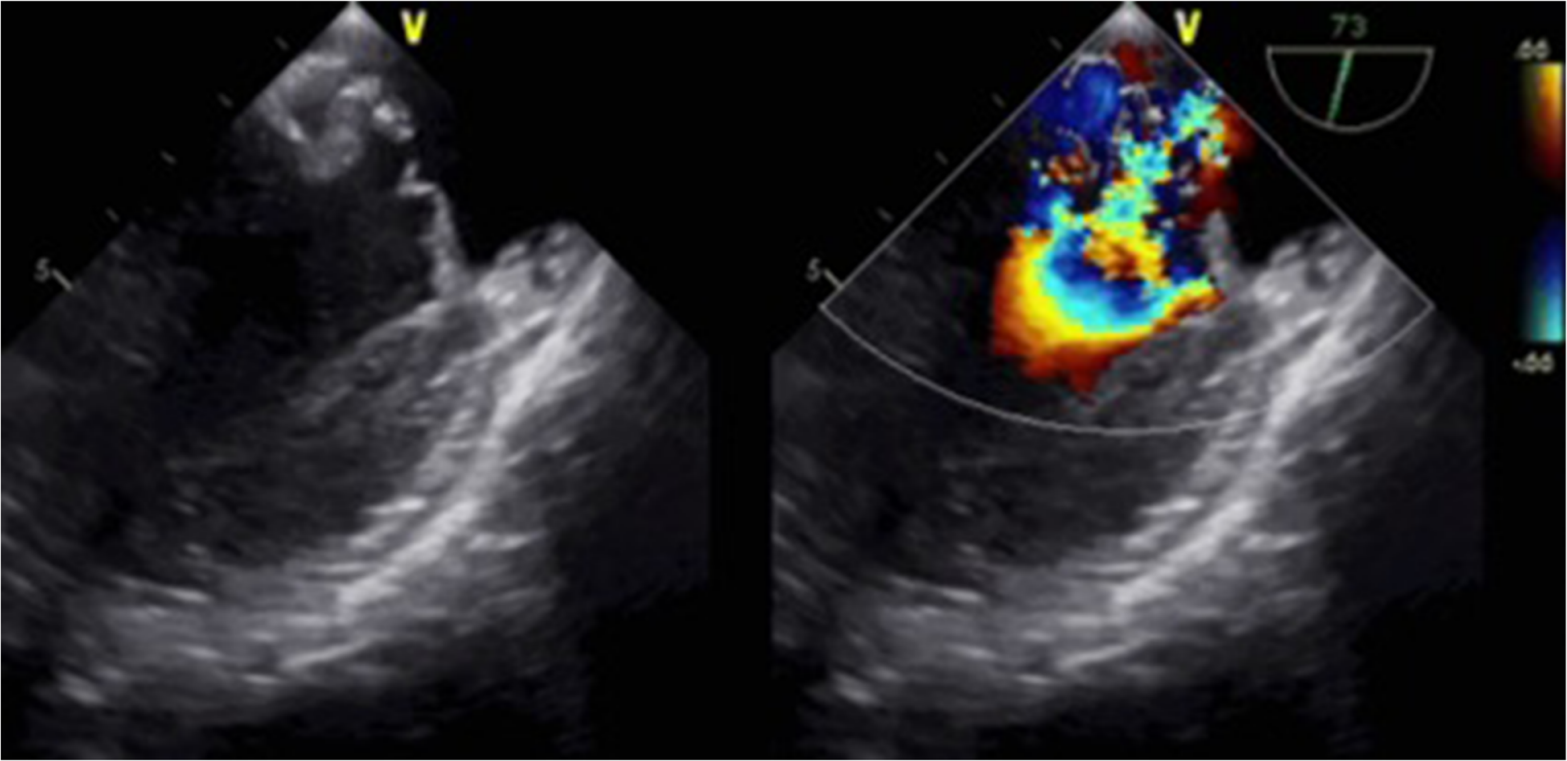
Transesophageal echocardiography showed acute mitral regurgitation and mitral valve tendon rupture


The patient was treated with blood pressure lowering medication and was admitted to the depressurized room in the intensive care unit; however, when the patient was placed in the supine position for central venous catheter insertion, his oxygenation worsened and he could not maintain his blood pressure. After tracheal intubation, the cause of the shock was investigated. When an echocardiogram with Doppler was performed, a severe mitral valve regurgitation (MR) was revealed (Fig. 3, Supplementary Material 1). In addition, based on transesophageal echocardiography, the cause of acute MR and respiratory failure was determined to be mitral valve tendon rupture (Fig. 4, Supplementary Material 2). The first polymerase chain reaction (PCR) of severe acute respiratory syndrome coronavirus 2 (SARS-CoV-2) was negative.

**Additional file 1: Supplementary Material 1**. Transthoracic echocardiography with doppler revealed a severe mitral valve regurgitation from the apical four chamber view.

The patient was referred to cardiovascular surgery, and extracorporeal membrane oxygenation was administered to maintain his hemodynamics. Due to high suspicion of COVID-19, two negative PCR are required. On the third day after admission, mitral valvuloplasty was performed. Surgical findings showed no evidence of endocarditis in the mitral valve, and no bacteria were cultured from blood or tissue cultures. Therefore, the patient was diagnosed with idiopathic mitral chordae tendineae rupture. He was discharged from the hospital 14 days after surgery, without complications.

## Discussion and conclusions

Acute MR is one of the causes of flash pulmonary edema leading to severe respiratory failure, which mimics acute hypertensive heart failure [6]. The sudden increase in left atrial pressure caused by acute MR increases back-flow to the pulmonary circulation, resulting in pulmonary edema. Chest radiography revealed bilateral symmetrical opacities in the central zones of the lung along with a butterfly shadow. EF is often preserved and tachycardia can be caused to compensate for the low effective blood flow to the ventricles as the blood flows easily back into the atrium. From a pathophysiological standpoint, the clinical presentation of acute MR is the one of rare causes of acute decompensated heart failure (ADHF) [7]. However, because acute MR often requires cardiac surgery and mechanical support, it is important that acute MR be differentiated from ADHF for timely intervention [8]. In addition, the diagnostic delay of acute MR increases mortality [8].

During the COVID-19 pandemic, the infiltrative shadow on the chest X-ray tends to lead to suspicion of COVID-19. This estimation could lead to premature closure, which leads to diagnostic error. In addition, acute MR often presents atypical chest radiographic findings such as pneumonia, as unilateral pulmonary edema, and infiltrative shadows along the trachea [9]. Precise auscultation and echocardiography using Doppler imaging play key roles in diagnosing acute MR; however, these two diagnostic tests are difficult to perform in areas restricted for infection control as physicians are wearing full PPE [10].

It has been reported that diagnostic errors during the COVID-19 pandemic can be classified into nine categories [11]. This case is classified as a diagnostic error due to anchoring bias, in which COVID-19 was suspected and accurate diagnosis was delayed [12]. The major reasons for this bias are the high probability of COVID-19 during pandemic and the insufficient examination to be performed due to infection control practices [11, 13].

To avoid the diagnostic delay of acute mitral regurgitation, a thorough history and physical examination are still important for the correct diagnosis and timely intervention of acute MR [14]. In addition, it is important to create an environment where auscultation can be performed with as little concern for infection as possible. Stethoscopes dedicated to one room—one patient is one of the solutions. Moreover, technology supports could be a solution to preventing anchoring bias during the pandemic, as a digital stethoscope and cable-less echography could be used to evaluate patients without concerns about contact infection.

In summary, COVID-19 could be an anchoring bias for emergency physicians evaluating patients with dyspnea, and various technological supports could provide an effective solution to avoid this kind of diagnostic delay. Furthermore, when acute MR is the diagnosis in otherwise healthy people, physicians should explore the cause of MR, such as acute myocardial infarction with chordae rupture, trauma, endocarditis, and long standing mitral valve prolapse.

## Supplementary Information

**Additional file 2: Supplementary Material 2**. Transesophageal echocardiography showed acute mitral regurgitation and mitral valve tendon.

## Data Availability

Not applicable.

## Abbreviations

COVID-19: Coronavirus disease 2019
MR: Mitral regurgitation
ED: Emergency department
PPE: Personal protective equipment
POCUS: Point of care ultrasound
EF: Ejection fraction
ECG: Echocardiogram
PCR: Polymerase chain reaction
SARS-CoV-2: Severe acute respiratory syndrome coronavirus 2
ADHF: Acute decompensated heart failure
